# Application of Chromosomal Microarray and Multiplex Ligation-dependent Probe Amplification in prenatal diagnosis

**DOI:** 10.1186/1755-8166-7-S1-P127

**Published:** 2014-01-21

**Authors:** Pankaj Sharma, Madhumita Roy Chowdhury, Neerja Gupta, Rashmi Shukla, Shruthi Sudarshan, Manju Ghosh, Deepika Deka, Madhulika Kabra

**Affiliations:** 1 Genetics Unit, Department of Pediatrics, All India Institute of Medical Sciences, New Delhi, India; 2 Department of Obstetrics & Gynecology, All India Institute of Medical Sciences, New Delhi, India

## Background

Chromosomal Microarray (CMA) and Multiplex Ligation-dependent Probe Amplification (MLPA) are relatively newer techniques for detecting cryptic copy number variations (CNVs). Here we are presenting the data of eight families where CMA and MLPA were used for prenatal diagnosis (PND) in view of their previous child with Intellectual disability/ developmental delay (ID/DD) and normal karyotype.

## Methods

Families with ID/DD children were referred for genetic counseling in Genetics Clinic, Department of Pediatrics, AIIMS. CNVs were studied in the probands using Illumina Cyto-SNP12 chips and MLPA kits for subtelomeric screening and microdeletion syndrome (P036 and P064) and PND by chorionic villus biopsy or amniotic fluid was performed in cases with pathogenic CNVs.

## Results

In eight families, CNVs of clear pathogenic significance were identified using CMA and confirmed by MLPA in seven cases. The probands had 2q32.3q33.3, 22q11.2, deletion in interstitial region while subtelomeric CNVs were 9q34 deletion, 8p deletion and 12p duplication, 5p deletion and 8q duplication in two affected sibs, 7q deletion and 10q duplication, 5p deletion and 6q duplication, 7q deletion and 20p duplication respectively. PND by MLPA (six cases) and CMA(two cases) were done. In six cases, fetus was found to be normal. In one case fetus had 8p deletion and 12p duplication while in another, the fetus had 6q deletion and 5p duplication.

## Discussion

CMA and MLPA have clearly demonstrated an increased resolution and improved detection rate of CNVs. CMA detects CNVs across the genome at high resolution enabling precise breakpoints of the CNVs. MLPA is a cost effective technique for developing countries but only limited (upto 45) nucleic acid targets in the genome can be investigated. In our settings it is an efficient technique for confirmation of CNVs and PND.

